# Prevention and management of physical health conditions in adults with severe mental disorders: WHO recommendations

**DOI:** 10.1186/s13033-021-00444-4

**Published:** 2021-03-03

**Authors:** Petra C. Gronholm, Neerja Chowdhary, Corrado Barbui, Jayati Das-Munshi, Kavitha Kolappa, Graham Thornicroft, Maya Semrau, Tarun Dua

**Affiliations:** 1 Department of Mental Health and Substance Use, World Health Organization, Geneva, Switzerland; 2 Centre for Global Mental Health and Centre for Implementation Science, Health Service and Population Research Department, Institute of Psychiatry, Psychology & Neuroscience, King’s College London, London, UK; 3 Department of Neuroscience, Biomedicine and Movement Sciences, Section of Psychiatry, WHO Collaborating Centre for Research and Training in Mental Health and Service Evaluation, University of Verona, Verona, Italy; 4 Department of Psychological Medicine, Institute of Psychiatry, Psychology & Neuroscience, King’s College London, London, UK; 5 The Chester M. Pierce, MD Division of Global Psychiatry, Massachusetts General Hospital, Boston, USA; 6 Brighton and Sussex Medical School, Centre for Global Health Research, Brighton, UK

**Keywords:** Severe mental illness, Schizophrenia, Bipolar affective disorders, Depression, Life expectancy, Mortality, Ethnicity, Deprivation, Schizoaffective disorders, Serious mental illness

## Abstract

**Background:**

People with severe mental disorders (SMD) experience premature mortality mostly from preventable physical causes. The World Health Organization (WHO) have recently produced guidelines on the prevention and management of physical health conditions in SMD. This paper presents the evidence which led to the recommendations presented in the guidelines.

**Methods:**

The work followed the methodological principles for WHO guideline development. Systematic reviews in relation to the treatment of seven key priority physical health conditions and associated risk factors in persons with SMD were systematically sourced. The quality of this evidence was assessed, and compiled into evidence profiles. Existing guidelines and treatment recommendations were also considered. Based on this information, specific recommendations were developed on the prevention and management of physical health conditions and their risk factors amongst people with SMD.

**Results:**

Nineteen recommendations were made in relation to the seven key priority physical health conditions and risk factors, alongside best practice statements for each condition. A mixture of conditional and strong recommendations were made, the quality of evidence underpinning these was generally low or very low. This is owing to the dearth of direct evidence relating to people living with SMD and comorbidities.

**Conclusions:**

This paper presents evidence-based recommendations to prevent and manage physical health conditions in people with SMD. The recommendations are designed to inform policy makers, healthcare providers as well as other stakeholders about what they can do to improve the management of physical health conditions in adults with SMD and support the promotion of individual health behaviors to reduce the risk factors for these conditions. If implemented, these recommendations can improve the care that people with SMD receive for their physical health conditions in an equitable and person-centered manner, so that in future in relation to premature mortality ‘no-one is left behind’.

## Background

The global burden of disease associated with mental disorders and addictive disorders was reported to be 7% as measured in disability-adjusted life-years (DALYs) in 2016 [1], with severe mental disorders (SMD) (i.e. schizophrenia and related conditions, bipolar disorder and moderate to severe depression) specifically affecting over 4% of the adult population worldwide [2]. This impact may also be underestimated due to the challenges in measuring the burden of mental disorders, and mental disorders may actually be level with cardiovascular and circulatory diseases in terms of DALYs, that is, accounting for 13% of DALYs [3].

In addition to causing a large proportion of morbidity, mental disorders—especially SMD—are linked with poorer health outcomes and increased mortality.

People with SMD have a 2–3 times higher average mortality compared to the general population, reflecting a 10–20 year reduction in life expectancy [4–6]. This pattern of increased rates of mortality is observed in both high and low-income settings [7–11].

While some of this excess mortality among people with SMD is attributed to unnatural causes (accidents, homicide, or suicide), the majority of deaths amongst people with SMD are attributable to physical health conditions, both non-communicable (NCDs) and communicable [4]. People with SMD have approximately 1.5–3 times higher risk of cardiovascular morbidity and mortality when compared with the general population [12]. People with SMD also have higher rates of diabetes mellitus [13], with reports of a 2–threefold higher prevalence compared with the general population. Infectious diseases such as HIV/AIDS contribute further to the high rates of premature death amongst people with SMD, as do other infectious diseases such as tuberculosis and hepatitis B and C [14].

There are a number of contributing factors that increase the risk of non-communicable diseases (such as cardiovascular disease, diabetes, respiratory illnesses, and cancers) for people with SMD. Firstly, adverse health-related behaviors such as physical inactivity, unhealthy diets, tobacco consumption, and alcohol use are more prevalent in people with SMD [15, 16]. Additionally, iatrogenic effects of many psychotropic medications used to treat the symptoms of SMD are also associated with an increased risk of developing physical health conditions and associated complications [12, 17]. Moreover, a growing body of research is illuminating the pathophysiological links between brain and body illness, via the stress cascade. Chronic stress can lead to blunted hypothalamic–pituitary–adrenal axis activity and inflammatory dysregulation throughout the body, which is associated with the development of both mental and physical illnesses [18, 19]. One well-known example is the impact of chronic stress on cardiovascular disease and the incidence of cardiovascular events [20, 21].

Overall, equitable access to comprehensive health services remains out of reach for the majority of people with SMD. People with SMD often lack access to health services or receive poor quality care, including promotion and prevention, screening, and treatment [22]. Socioeconomic disadvantages and stigma and discrimination associated with SMD may further influence the health and health care amongst this population [23]. Most studies reporting on excess mortality in people with SMD are from high income countries. The situation may be worse in low-income settings where resources are inadequate, institutions are not well managed and access to quality mental health care and physical care is limited [6, 11].

It is crucial to address the disparities in health care access and provision for people with SMD, and develop comprehensive evidence-based clinical guidelines that provide recommendations to support the scale-up of care for physical health conditions and their risk factors affecting people living with SMD globally.

This paper is based on the WHO Guidelines on Management of Physical Health Conditions in Adults with Severe Mental Disorders (PH-SMD) [24], and presents recommendations to health care workers at all levels of the health care system, and other stakeholders, on how to prevent and manage comorbid physical and mental health conditions.

## Methodology for the development of guidelines

These global evidence-informed recommendations were developed by WHO, following a rigorous guideline development methodology [25] and the GRADE (Grading of Recommendations Assessment, Development and Evaluation) approach [26, 27].

The process started with an initial phase to identify the key conditions and associated risk factors that would be included in the guidelines. A scoping review was conducted of conditions and risk factors that increase the morbidity and mortality for people with SMD. This scoping review involved three stages: (i) an initial broad focus on risk factors interventions for excess mortality and morbidity in people with SMD, guided by previous work exploring epidemiology and risk factors for excess mortality in persons with SMD [28] and the physical health conditions and risk factors identified in this report; (ii) review of existing WHO guidelines, and (iii) findings of a WHO consultation on the topic, and other relevant WHO documents and discussions with the WHO steering group. The scoping review results were considered in a consultation with the Guideline Development Group (GDG), reflecting externally appointed international experts convened by the WHO. Following this review and consultation, seven key priority health conditions and risk factors were identified: tobacco use disorder, overweight/obesity, substance use disorder, cardiovascular disease and cardiovascular risk, diabetes mellitus, HIV/AIDS, and other infectious diseases (Tuberculosis, Hepatitis B/C). These conditions and risk factors are not considered a comprehensive list of physical health conditions, but were considered to reflect important conditions shown to increased morbidity and mortality for people with SMD, and for which there was evidence available.

A priori research questions were formulated for these conditions and risk factors, following the PICO format (Population, Intervention, Comparison group, Outcomes). These questions were ratified by the WHO Guideline Review Committee, to define the final specific research questions regarding the physical health conditions and risk factors that would be addressed in the final disseminated guidelines. These specific PICO questions were addressed through comprehensive searches of systematic review evidence. High quality systematic reviews were identified through the Cochrane Library (including DARE), PubMed/Medline, Embase, Psycinfo, Epistemonikos, and the Global Health Library; the searches for each question were formulated informed by consultations with guideline methodologists and subject experts at the WHO.

Following these searches, n = 23 systematic reviews (selected from well over n = 150 reviews) were identified that met the criteria of: (i) recency (published within the previous 5 years [i.e. 2013 onwards]); (ii) methodological quality (sufficiently high ratings [positive rating on more than 6 of 11 quality domains] on the Assessment of Multiple Systematic Reviews tool (AMSTAR) [29–31]); and (iii) relevance (evidence in reviews was closely relevant to the PICO population).

To ensure evidence-based recommendations for the guidelines were developed in a rigorous manner, the evidence identified through these searches was reviewed using the GRADE methodology [26, 27] by two independent raters. Information relevant to the PICO questions for each key condition and risk factor were extracted. In line with WHO principles for guideline development, certainty of included evidence was assessed via the GRADE approach, involving a quality assessment considering study design, risk of bias, inconcistencies, indirectness, imprecisions, and the reporting of bias in included studies. Based on this GRADE assessment, quality of the evidence was characterised as either high, moderate, low or very low.

Where relevant systematic review evidence could not be identified for a given condition or risk factor, other related guidelines and tools/resources were considered in the development of the recommendations. Some of these are detailed in Box 1. When these resources were considered, the process adopted to review the evidence entailed the following steps: (i) review of evidence that exists for the interventions to manage physical health conditions and associated risk factors in people with SMD; (ii) examination of the extent to which existing recommendations for the general population (especially from existing WHO guidelines) can be applied to people with SMD; (iii) examination of when and how these recommendations need to be adapted for people with SMD; and, (iv) to provide recommendations that are specific to this population when needed.

Final recommendations were developed via discussions with the GDG, guideline methodologists, the evidence review team, and the WHO secretariat. These discussions considered the background evidence for each PICO question; the GRADE evidence profiles and certainty of evidence; and specific aspects of the conditions and risk factors specified in the PICO questions (e.g. priority, anticipated effects, value attached to outcomes, cost effectiveness, impact on health equity, acceptability, and feasibility). The final recommendations that emerged from this report on both the certainty of evidence and the strenght of the recommendation.

The GRADE evidence tables and Evidence to Decision tables based on which recommendations are made are available in the guideline Evidence Profile document, available at the WHO website [32].

Box 1: Related WHO guidelines and tools 1. Mental Health Gap Action Programme (mhGAP) Intervention Guide for mental, neurological, and substance use disorders in non-specialized health settings (Version 2.0). Geneva, WHO, 2016. http://www.who.int/mental_health/mhgap/mhGAP_intervention_guide_02/en/

2. Package of Essential Noncommunicable (PEN) Disease Interventions for Primary Health Care in Low-Resource Settings. Geneva, WHO, 2010. http://www.who.int/cardiovascular_diseases/publications/pen2010/en/

3. Strengthening health systems for treating tobacco dependence in primary care. Building capacity for tobacco control: training package. http://www.who.int/tobacco/publications/building_capacity/training_package/treatingtobaccodependence/en/

4. Global recommendations on physical activity for health. Geneva, WHO, 2010. http://www.who.int/dietphysicalactivity/publications/9789241599979/en/

5. Consolidated guidelines on HIV prevention, diagnosis, treatment, and care for key populations. Geneva, WHO, 2016 update. http:// www.who.int/hiv/pub/guidelines/keypopulations-2016/en/

6. Consolidated guidelines on the use of antiretroviral drugs for treating and preventing HIV infection. Recommendations for a public health approach. Second edition. Geneva, WHO, 2016. http://www.who.int/hiv/pub/arv/arv-2016/en/

7. WHO guidelines for the treatment of drug-susceptible tuberculosis and patient care (2017 update) https://www.who.int/tb/publications/2017/dstb_guidance_2017/en/

8. WHO consolidated guidelines on drug-resistant tuberculosis treatment https://www.who.int/tb/publications/2019/consolidated-guidelines-drug-resistant-TB-treatment/en/

9. Guidelines for the prevention, care, and treatment of persons with chronic hepatitis B infection. Geneva, WHO, 2015. https://www.who.int/hepatitis/publications/hepatitis-b-guidelines/en/

10. Guidelines for the screening, care, and treatment of persons with chronic hepatitis C infection. Geneva, WHO, 2016. http://www.who.int/hepatitis/publications/hepatitis-c-guidelines-2016/en/

## Results

### WHO recommendations: management of physical health conditions in adults with severe mental disorders

The primary audience for the WHO Guidelines on Management of Physical Health Conditions in Adults with SMD (PH-SMD) is health care workers providing services for people with SMD at all levels of the health care system, including outpatient and inpatient care at first-level, second-level, district and tertiary healthcare facilities. In addition, these guidelines are of interest to policy makers and health care planners at the national and local levels, national and regional mental health programme managers, national and regional primary care programme managers, members of national and local health departments, people living with SMD and their families, and groups representing people with SMD and their families.

Recommendations are made for the management of the seven physical health conditions and risk factors that increase the morbidity and mortality for people with SMD, and that were prioritized in the WHO guidelines. The specific recommendations made based on the evidence reviewed for these guidelines are presented in Box 2.

The guidelines additionally include best practice statements that represent important considerations to support implementation of the recommendations.

Furthermore, information has been presented for drug-drug interactions between medicines relevant for the physical health conditions and medicines used for SMD. Searches between both lists (medicines relevant for the physical health conditions and medicines used for SMD) were conducted using the drug-drug interaction software Lexi-Interact [33]. Lexi-Interact was chosen as it is commonly used in clinical practice and scored well on accuracy and comprehensiveness in a recent review comparing five drug-drug interaction engines [34].

Box 2: WHO-PH-SMD Guidelines recommendations **Tobacco use disorder (in the context of tobacco cessation programmes)**

Recommendation 1: In people with severe mental disorders, combined pharmacological and non-pharmacological interventions may be considered in accordance with the WHO training package (*Strengthening health systems for treating tobacco dependence in primary care. Building capacity for tobacco control: training package*). *(Strength of recommendation: Conditional; quality of evidence: Very low).*

Recommendation 2: In people with severe mental disorders, a directive and supportive behavioral intervention programme may be considered and should be tailored to the needs of the population. *(Strength of recommendation: Conditional; quality of evidence: Very low).*

Recommendation 3: In people with severe mental disorders, varenicline, bupropion and nicotine replacement therapy may be considered for tobacco cessation. *(Strength of recommendation: Conditional; quality of evidence: Very low).*

Best practice statement*:* Prescribers should take into account potential interactions between buproprion and varenicline with psychotropic medications as well as possible contra-indications.

**Overweight/obesity**

Recommendation 1: Behavioral lifestyle (healthy diet, physical activity) interventions should be considered in all people with severe mental disorders who are overweight or obese or at risk of becoming overweight or obese in accordance with *WHO’s Package of Essential Noncommunicable Disease Interventions (WHO PEN) for primary care in low-resource settings (2010)*. These interventions should be appropriate and tailored to the needs of this population. *(Strength of recommendation: Strong; Quality of evidence: Very low).*

Recommendation 2: For people with severe mental disorders who are overweight or obese, and where lifestyle interventions and/or switching psychotropic medication do not appear successful, adjunctive metformin may be considered. This should be considered under close clinical supervision and monitoring. *(Strength of recommendation: Conditional; Quality of evidence: Low).*

Best practice statements:

- For people with severe mental disorders who are overweight or obese or at risk of becoming overweight or obese, initiating a psychotropic medication with lower propensity for weight gain should be considered, taking into account clinical benefits and potential adverse effects.
- For people with severe mental disorders who are overweight or obese, switching to a psychotropic medication with a lower propensity for weight gain may be considered, taking into account clinical benefits and potential adverse effects.

**Substance use disorders**

Recommendation 1: For people with severe mental disorders and comorbid substance use disorders (drug and/or alcohol), interventions should be considered in accordance with the WHO mhGAP guidelines. *(Strength of recommendation: Conditional; Quality of the evidence: Low).*

Recommendation 2: Non-pharmacological interventions (e.g. motivational interviewing) may be considered and tailored to the needs of people with severe mental disorders and substance use disorders *(Strength of recommendation: Conditional; Quality of the evidence: Very low).*

Best practice statement: Prescribers should take into account the potential for drug-drug interactions between medicines used for treatment of substance use disorders and severe mental disorders.

**Cardiovascular disease and cardiovascular risk**

Recommendation 1: For people with severe mental disorders and pre-existing cardiovascular disease, or with cardiovascular risk factors (e.g. high blood pressure or high cholesterol), pharmacological and non-pharmacological interventions may be considered in accordance with the *WHO Package of Essential Noncommunicable Disease Interventions (WHO PEN) for primary care in low-resource settings (2010)* for lowering cardiovascular risk and management of cardiovascular disease. *(Strength of recommendation: Strong; Quality of evidence: High to moderate for different interventions).*

Recommendation 2: For people with severe mental disorders and pre-existing cardiovascular disease, the following is recommended:

a) Behavioral lifestyle (healthy diet, physical activity) interventions may be considered. These interventions should be appropriate and tailored to the needs of this population. *(Strength of recommendation: Conditional; Quality of evidence: Very low).*

b) Collaborative care i.e. a multi-professional approach to patient care with a structured management plan, scheduled patient follow-up, and enhanced inter-professional communication, may be considered for cardiovascular disease management. *(Strength of recommendation: Conditional; Quality of evidence: Very low).*

Recommendation 3: For people with severe mental disorders and cardiovascular risk factors, behavioral lifestyle (healthy diet, physical activity) interventions may be considered. These interventions should be appropriate and tailored to the needs of this population. *(Strength of recommendation: Conditional; Quality of evidence: Very low).*

Best practice statements: For people with severe mental disorders and pre-existing cardiovascular disease:

- Initiating a psychotropic medication with lower propensity for cardiovascular risk is a strategy that should be considered, taking into account clinical benefits and potential adverse effects.
- Switching to a psychotropic medication with lower propensity for cardiovascular risk may be considered, taking into account clinical benefits and potential adverse effects.
- For people with severe mental disorders and pre-existing cardiovascular disease or cardiovascular risk factors:
- Prescribers should be aware of potential interactions between prescribed medicines for cardiovascular disease and prescribed psychotropic medications, which may affect cardiovascular risk. Cardiovascular outcomes and risk factors should be monitored and dose adjustment of cardiovascular medicines may be required.

**Diabetes mellitus**

Recommendation 1: For people with severe mental disorders and diabetes mellitus, interventions in accordance with the WHO Package of Essential Non-communicable (PEN) Disease Interventions for Primary Health Care in Low-Resource Settings should be considered for diabetes management.*(Strength of recommendation: Strong; Quality of evidence: Low).*

Recommendation 2: Behavioral lifestyle interventions should be considered for all people with severe mental disorders and diabetes mellitus. These interventions should be appropriate and tailored to the needs of this population. *(Strength of recommendation: Strong; Quality of evidence: Very low).*

Recommendation 3: In people with depression and comorbid diabetes mellitus, cognitive behavior therapy for treatment of depression may be considered. *(Strength of recommendation: Conditional; Quality of evidence: Very low).*

Best Practice Statements: For people with severe mental disorders and diabetes mellitus:

- Initiating an anti-psychotic medication with lower propensity for producing hyperglycaemia should be considered, taking into account clinical benefits and potential adverse effects.
- Switching to an anti-psychotic medication with lower propensity for producing hyperglycaemia is a strategy that may be considered, taking into account clinical benefits and potential adverse effects.
- Prescribers should be aware of potential interactions between prescribed medicines for diabetes mellitus and prescribed psychotropic medicines, which may affect glycaemic control. Glycaemic control should be monitored and dose adjustment of medicines may be required.

**HIV/AIDS**

Recommendation 1: For people with severe mental disorders and HIV/ AIDS, antiretroviral drugs should be considered in accordance with the *WHO Updated recommendations on first-line and second-line antiretroviral regimens. (Strength of the recommendation: Strong; Quality of the evidence: Moderate).*

Recommendation 2: Additional psychosocial support for treatment adherence should be provided to people with HIV and severe mental disorders in accordance with the *WHO consolidated guidelines on the use of antiretroviral drugs for treating and preventing HIV infection. (Strength of the recommendation: Strong; Quality of the evidence: Moderate).*

Best practice statement**:** for people with severe mental disorders and HIV/ AIDS, prescribers should take into account the potential for drug-drug interactions between antiretroviral drugs and psychotropic medicines.

**Other infectious diseases (Tuberculosis, Hepatitis B/C)**

Recommendation 1: For people with severe mental disorders and TB, pharmacological management should be considered in accordance with the *WHO guidelines for the treatment of drug-susceptible tuberculosis and patient care* and the *WHO treatment guidelines for drug-resistant tuberculosis. (Strength of the recommendation: Strong; Quality of the evidence: Low).*

Recommendation 2: For people with severe mental disorders and TB, non-pharmacological (social, psychological) management should be considered in accordance with the *WHO guidelines for the treatment of drug-susceptible tuberculosis and patient care* and the *WHO treatment guidelines for drug-resistant tuberculosis. (Strength of the recommendation: Strong; Quality of the evidence: Low)*.

Recommendation 3: For people with severe mental disorders and Hepatitis B, treatment should be considered in accordance with the *WHO guidelines for the prevention, care and treatment of persons with chronic Hepatitis B infection. (Strength of the recommendation: Strong; Quality of the evidence: Low).*

Recommendation 4: For people with severe mental disorders and Hepatitis C, treatment should be considered in accordance with the *WHO guidelines for the screening care and treatment of persons with chronic Hepatitis C infection*. *(Strength of the recommendation: Strong; Quality of the evidence: Low).*

Best practice statement: For people with severe mental disorders and TB and/or Hepatitis B/, prescribers should take into account the potential for drug-drug interactions between TB medicines, medicines for Hepatitis B and C with psychotropic medicines.

## Discussion

### Guideline implementation considerations

The recommendations in the WHO guidelines must be implemented using a person-centered and integrated approach to address factors associated with increased morbidity and mortality in people with SMD. This integration is needed at four levels: screening and early detection of physical health conditions, counselling for behavioral risk factors, assessment and management of cardiovascular disease risk, and management of established physical and mental health conditions.

We propose a multilevel intervention framework that will be useful for designing, implementing and evaluating interventions and programmes to reduce excess mortality in people with SMD [4]. The first level is individual-focused interventions. The second and third levels of the framework consist of strategies focused on the health systems and socio-environmental context, respectively, which provide the enabling environment for implementation of the recommendations.

1. The individual-focused interventions include an enabling environment to follow the recommendations with support to programme managers and health care practitioners in the use of evidence-based practices.
2. The next level in the framework encompasses strategies within health systems to strengthen them such as care coordination and increased workforce.
3. The broadest level of the framework incorporates socioenvironmental factors and includes community interventions such as peer and family support programmes and stigma reduction programs as well as policy-level interventions that ensure parity for mental health.

### Limitations of the guideline development process

The strength of these guidelines lies in the transparent and robust methodologies that were applied for evidence synthesis, and the development of recommendations according to the WHO Handbook for Guideline Development [25, 35]. However, these guidelines have some limitations.

Developing recommendations for physical health conditions and associated risk factors in people with SMD within the formal WHO guideline development framework highlighted several challenges, arising mainly from the extent of the available evidence. Much of the evidence came from well-resourced settings which could potentially impact on generalizability. For example, malnutrition in SMD was anecdotally highlighted as a concern in some low- and middle-income settings (as opposed to obesity), yet no studies meeting inclusion criteria were identified on this important issue.

One of the most challenging aspects of the process was generalizing evidence developed for the general population to people with SMD. While general population interventions geared towards NCDs, infectious diseases or other health problems are likely to be as effective for persons with SMD, given the special needs of this population, interventions for SMD may require tailoring. However, the evidence for this was limited. In instances where the evidence was limited, we took advantage of the GRADE methodology [26, 27], which clearly recognizes that, in addition to the evidence base, other aspects that are expected to inform the recommendations include consideration of values such as gender equality, equity and the protection of human rights, feasibility, resource use and the knowledge and experience of the Guideline Development Group experts.

Another limitation of these guidelines is that, given feasibility constraints, they are based on evidence from existing systematic reviews. Although these reflect the most recent evidence, this approach may have led to omission of more recent individual studies not included in these reviews. Also, people with SMD may have more than one physical health condition or risk factor. However, the available evidence for these guidelines is only focused on individual health conditions. This means these guidelines do not examine multiple co-morbidities, albeit tackling multimorbidity is recognized as a key global health challenge [36].

Since the publication of these guidelines, the SARS-CoV-2 (COVID-19) pandemic represents a further condition which warrants particular attention in relation to people with SMD [37, 38]. It seems likely that COVID-19 will exacerbate risk factors for excess mortality amongst this population, albeit the extent of this impact is not yet clear. Measures are called for to safeguard the physical health of people with SMD specifically during the COVID-19 pandemic, for example through individual support with infection prevention alongside health promotion strategies such as the ones presented in these PH-SMD guidelines, and continued access to contact with mental healthcare teams and reviews of mental health needs [39]. Future research and knowledge gaps.

The guideline development process highlighted a relative dearth of evidence relating to people living with SMD and comorbidities. While evidence from the general population on comorbidities may be tailored to SMD populations, there is potentially a need for specific interventions that have been developed with SMD populations in mind. Another important area of research will be to assess the effects of health system and policy interventions on excess mortality in SMD. We need to understand why those with SMD have not benefitted from trends in the general population towards reduced mortality in certain diseases and smoking cessation. Researchers should take advantage of natural experiments, and design studies in health systems and at the population level to evaluate the impact of these programs.

Many people with SMD have multiple cardiovascular and other risk behaviors which may be modifiable, and future research should test interventions addressing multiple risk factors, as well as those which are directly linked to mortality.

Cost-effectiveness models of different approaches in people with SMD are important, especially in low resource settings, however there was very little evidence identified relating to this important issue. Future research in this area will be important as we aim to achieve universal health coverage and to address the physical health needs of this vulnerable population.

Research is needed to identify and manage barriers to and facilitators of implementing evidence-based guidance and policy recommendations. We need to understand how to deliver evidence-based interventions successfully in the real world, taking into account training and workforce issues, and the often-limited resources in local community settings. We need to understand to what extent interventions and programs could or should be disseminated across countries.

Finally, to gain a better understanding of the different perspectives involved, qualitative research is needed to understand the experiences of users, providers, family members, as well as professionals’ receptivity to education and training.

## Conclusions

In this paper we present evidence-based recommendations to manage physical health conditions and associated risk factors in people with SMD. The recommendations are designed to inform policy makers, healthcare providers as well as other stakeholders about what they can do to improve the management of physical health conditions in adults with SMD and support the promotion of individual health behaviors to reduce the risk factors for these conditions.

By decreasing morbidity and premature mortality amongst people with SMD, these guidelines will help achieve target 3.4 of the United Nations Sustainable Development Goals (“By 2030, reduce by one third premature mortality from non-communicable diseases through prevention and treatment and promote mental health and well-being”) [40] and facilitate the implementation of WHO’s Comprehensive Mental Health Action Plan [41].

These guidelines will be reviewed in three to five years, unless an earlier review and update is warranted by breakthrough research. New evidence in these areas is regularly monitored by WHO, in consultation with international experts and academic partners. We also expect revisions to include a review of relevant new questions or areas currently not covered by these guidelines.

We intend that the publication of the WHO guidelines, and the evidence-base that underpins them, will stimulate interest in the global scientific, policy and practitioner communities in strengthening evidence where it is weak. We also hope that the guidelines will improve the care that people with SMD receive for their physical health conditions in an equitable and person-centered manner, so that in future in relation to premature mortality ‘no-one is left behind’.

## Data Availability

Data sharing is not applicable to this article as no datasets were generated or analysed during the current study. All materials based on which these guideline recommendations are made are publicly available.

## Abbreviations

DALYs: Disability-adjusted life-years
GRADE: Grading of Recommendations Assessment, Development and Evaluation methodology
NCDs: Non-communicable diseases
PH-SMD: WHO Guidelines on Management of Physical Health Conditions in Adults with Severe Mental Disorders
SMD: Severe mental disorders
WHO: World Health Organization
